# Effect of Transcranial Direct Current Stimulation on Memory and Emotional Recovery in Patients with Stroke and Traumatic Brain Injury: A Prospective, Multicenter, Interventional Pilot Study

**DOI:** 10.3390/jcm14062083

**Published:** 2025-03-19

**Authors:** Marta Szczepańska, Zofia Twardochleb, Maciej Miś, Marcin Miś, Adam Druszcz, Małgorzata Paprocka-Borowicz, Joanna Rosińczuk

**Affiliations:** 1 Centre of Neurorehabilitation AFA-MED, 91-829 Lodz, Poland; szczepanska.marta@op.pl (M.S.); z.twardochleb@wp.pl (Z.T.); 2 Department of Neurosurgery, University Centre of Neurology and Neurosurgery, Wroclaw Medical University, 50-556 Wroclaw, Poland; mis.neurochirurg@gmail.com (M.M.); marcin.mis@gmail.com (M.M.); 3 Faculty of Medicine, Wrocław University of Science and Technology, 50-376 Wroclaw, Poland; dr.druszcz@gmail.com; 4 Department of Neurosurgery, Provincial Specialist Hospital in Legnica, 59-220 Legnica, Poland; 5 Division of Clinical Physiotherapy and Rehabilitation, University Centre of Physiotherapy and Rehabilitation, Faculty of Physiotherapy, Wroclaw Medical University, 55-355 Wroclaw, Poland; malgorzata.paprocka-borowicz@umw.edu.pl; 6 Department of Neurological Rehabilitation, Regional Specialist Hospital in Wroclaw, 51-128 Wroclaw, Poland; 7 Department of Nursing, Faculty of Nursing and Obstetrics, Wroclaw Medical University, 51-618 Wroclaw, Poland

**Keywords:** transcranial direct current stimulation, ischemic stroke, traumatic brain injury, neurorehabilitation, cognitive recovery, emotional states, depression, memory

## Abstract

**Background/Objectives:** Emotional and cognitive impairments are prevalent in patients with acute ischemic stroke (AIS) and traumatic brain injury (TBI), significantly affecting their quality of life and recovery potential. Transcranial direct current stimulation (tDCS) has emerged as a promising non-invasive method to enhance neurorehabilitation outcomes by modulating neural activity. **Methods:** This prospective, open-label, multicenter interventional study included 100 participants (50 AIS, 50 TBI) who underwent 10 sessions of tDCS. Emotional states, depression levels, and memory and learning outcomes were assessed pre- and post-intervention using the UWIST Mood Adjective Checklist (UMACL), Depression Measurement Questionnaire (DMQ), Benton Visual Retention Test (BVRT), and Brain Damage Diagnostic Test (BDDT). **Results:** Significant improvements in emotional states were observed post-tDCS. Hedonic tone increased (AIS: 2.5 to 5 stens; TBI: 1.5 to 4 stens), while tension arousal decreased (AIS: 8 to 6 stens; TBI: 8 to 6 stens; all *p* < 0.001). Depression levels dropped significantly, with the overall depression index decreasing from 131 to 100 points in AIS and from 126 to 104 points in TBI (both *p* < 0.001). Memory and learning scores improved significantly, evidenced by increased correct responses and reduced errors in BVRT and BDDT tests (all *p* < 0.001). **Conclusions:** tDCS effectively improved emotional states, reduced depression levels, and enhanced cognitive functions in AIS and TBI patients. These findings support the integration of tDCS into neurorehabilitation protocols, with further research needed to explore long-term benefits and individualized treatment strategies.

## 1. Introduction

According to recent reviews, both acute ischemic stroke (AIS) and traumatic brain injury (TBI) are driven by multifaceted cellular and molecular cascades that can lead to irreversible neurological deficits [1,2]. In AIS, the sudden compromise of cerebral blood flow triggers excitotoxicity, mitochondrial dysfunction, and oxidative stress, while prolonged inflammatory responses further weaken the blood–brain barrier and disrupt neural circuits [1]. TBI, on the other hand, encompasses primary mechanical damage (e.g., contusions, axonal shearing) and secondary, longer-term processes such as microglial overactivation, Wallerian-like axonal degeneration, and an insufficient endogenous neuroregenerative response [2]. Ultimately, both conditions result in complex pathologies that include neuronal loss, glial scar formation, and limited repair capacity, underscoring the need for novel therapeutic strategies to support recovery and long-term functional improvement [1,2].

Both conditions are leading causes of disability worldwide, affecting millions of individuals annually [3]. Stroke alone is estimated to impact approximately 15 million people each year, with about 5 million survivors experiencing long-term impairments [4]. TBI, on the other hand, affects more than 60 million people globally each year, encompassing a wide spectrum of severity and clinical manifestations [5]. Both conditions result in significant disruptions to physical, cognitive, and emotional well-being, often leading to lifelong changes in daily functioning and quality of life.

Traditional rehabilitation strategies have primarily focused on physical recovery—such as improving motor function and activities of daily living—while the cognitive and emotional domains have sometimes been underrepresented or pursued through less standardized approaches. As a result, deficits in memory, attention, executive function, and emotional regulation frequently persist, underscoring the need for integrated, multimodal interventions [6,7].

Recent advances in neuromodulation techniques, such as transcranial direct current stimulation (tDCS), have garnered attention for their potential to enhance neuroplasticity and accelerate recovery in neurological disorders [8]. tDCS is a non-invasive brain stimulation method that delivers weak direct currents—typically in the range of 1–2 mA—through electrodes placed on the scalp. By modulating neuronal excitability, tDCS can facilitate or inhibit synaptic activity in targeted brain regions, thus creating a more favorable environment for neuroplastic changes [9].

Studies in stroke and TBI populations have demonstrated improvements in motor function, language, attention, and working memory, suggesting that tDCS can be broadly beneficial in neurorehabilitation [10,11]. Additionally, researchers have reported a reduction in neuropsychiatric symptoms, including depressive and anxiety-related disorders, further highlighting the technique’s therapeutic versatility [12,13,14].

One of the central mechanisms by which tDCS may exert its beneficial effects is through the enhancement of synaptic plasticity, a fundamental process underlying learning, memory, and behavioral adaptation. This mechanism has direct implications for rehabilitation: by shifting cortical excitability, tDCS could help maximize the impact of standard therapeutic exercises and cognitive training [15]. Beyond this, animal and human studies suggest that tDCS may influence neurotrophic factors [16], cerebral blood flow [17], and inflammatory pathways [18], all of which are critical to recovery and tissue repair following brain injury. However, despite these promising findings, routine clinical adoption of tDCS remains limited. Variations in study designs, stimulation intensity, electrode placements, patient characteristics, and outcome measures contribute to heterogeneous results, highlighting the need for more standardized research protocols.

It should be emphasized that neurorehabilitation is a multifaceted and rapidly evolving discipline that merges principles from neurology, neurosurgery, physical therapy, occupational therapy, psychology, and related fields to promote functional recovery in individuals with neurological disorders [19]. Whether the injury arises from vascular events or mechanical trauma, the overarching goal is to harness neuroplasticity—namely, the brain’s ability to reorganize neuronal pathways and establish new connections [20,21]—to improve daily functioning, cognition, and emotional well-being [22]. This approach involves a variety of techniques, including task-oriented training, cognitive remediation, psychosocial interventions, and increasingly, adjunctive neuromodulation methods such as tDCS [23]. By targeting shared pathophysiological mechanisms (e.g., excitotoxicity, neuroinflammation, maladaptive remodeling), neurorehabilitation often yields cross-cutting benefits in different neurological conditions, although each disorder’s unique injury profile may necessitate individualized protocols.

Therefore, we aim in this paper to highlight how a neurorehabilitation-focused intervention—like tDCS—can support both physical and neurocognitive recovery in populations with AIS and TBI, while also acknowledging the distinct etiological and clinical challenges posed by each condition. This study addresses these gaps by evaluating the effects of a standardized tDCS protocol combined with neurologopedic (speech) rehabilitation on memory and emotional recovery in AIS and TBI patients. Neurologopedic interventions focus on speech, language, and cognitive–communication disorders, but also encompass broader cognitive–linguistic functions. By systematically incorporating both tDCS and targeted cognitive–emotional training, this research aims to provide a clearer, evidence-based understanding of how to optimize recovery in these populations.

## 2. Materials and Methods

### 2.1. Study Design and Participants

This study was a prospective, open-label, multicenter interventional study conducted from May 2018 to February 2021. A group of 100 patients with neurological disorders following brain injury were assigned to two comparative groups: ischemic stroke (n = 50, AIS) and traumatic brain injury (n = 50, TBI). All study participants were patients of the specialized neurorehabilitation clinics AFA-MED in Żary, Szczecin, and Łódź (Poland), as well as patients who participated in therapeutic stays organized by AFA-MED in Międzywodzie (Poland).

### 2.2. Study Ethics

Participation in the research project was voluntary and anonymous. The research project received positive approval from the Bioethics Committee of the Wroclaw Medical University in Poland (KB–223/2018). All study participants were informed about the course of the study and provided written informed consent to participate. The study protocol was prepared in accordance with the requirements of the Declaration of Helsinki and Good Clinical Practice [24]. Each patient was informed about the possibility of withdrawing at any stage of the research.

### 2.3. Patients’ Enrollment

The criteria ensuring the inclusion of patients in the study were verified by a neurologist and a psychologist and included age over 18 years, a history of ischemic stroke or traumatic brain injury, absence of chronic conditions or mental disorders hindering participation in the study, no contraindications to tDCS sessions, obtaining voluntary and informed consent to participate in the study, and expressing voluntary and informed consent by the patient to participate in the study.

Patients were excluded if they were under 18 years of age, had no history of ischemic stroke or traumatic brain injury (or had brain injuries from other causes), exhibited cognitive impairments or mental conditions preventing independent completion of questionnaires, had other disorders hindering study participation, presented contraindications to tDCS sessions (e.g., implanted metal devices, pregnancy, active cancer, aneurysms, metastases, as well as poor general health, fatigue, fever, or cold symptoms), or did not provide voluntary and informed consent.

### 2.4. Screening and Recruitment

A total of 160 patients were initially screened for eligibility. Screening was conducted by a neurologist and psychologist based on the above-mentioned inclusion and exclusion criteria. Out of 160 patients, 60 were excluded for the following reasons: cognitive impairments (n = 20 were unable to independently complete questionnaires or assessments), medical contraindications to tDCS (n = 15 presented conditions such as pacemakers, metal implants, or uncontrolled epilepsy), other medical or psychiatric conditions (n = 10 were excluded due to severe psychiatric disorders or unrelated neurological disorders), and voluntary withdrawal (n = 15 declined to provide informed consent or decided not to participate before tDCS administration).

### 2.5. Research Procedure

A total of 100 patients were ultimately enrolled in the study and purposefully divided into two comparative groups: patients after ischemic stroke (n = 50, AIS) and patients after traumatic brain injury (n = 50, TBI). Both groups underwent an experimental intervention involving non-invasive brain macrostimulation—tDCS. Standard therapeutic procedures in the form of neurologopedic rehabilitation were guaranteed for both groups. All 100 enrolled participants completed the study protocol, including 10 sessions of tDCS and post-intervention assessments. No dropouts occurred during the intervention phase, and all participants’ data were included in the final analyses.

#### 2.5.1. Transcranial Direct Current Stimulation

For the tDCS procedures, a high-resolution, certified device meeting all quality requirements and safety standards was used. A dual-channel Soterix Medical tDCS stimulator (Soterix Medical Inc., Woodbridge, NJ, USA) compatible with the Soterix 4 × 1 HD-tES adapter (Soterix Medical Inc., Woodbridge, NJ, USA) [25] was applied. All tDCS procedures were conducted and supervised by a doctoral student with the necessary qualifications and experience, including training in neuropsychological diagnostics, EEG-based biofeedback (neurofeedback), and tDCS stimulation. The tDCS sessions were carried out in three panels over five months—during the first (I), third (II), and fifth (III) months, with daily sessions lasting 30 to 40 min for 5, 7, and 10 consecutive days, respectively. Table 1 shows a detailed breakdown of therapeutic panels, including individual sessions, stimulation parameters, and electrode placements.

#### 2.5.2. Standard Neurologopedic Rehabilitation

Each participant underwent an individualized neurologopedic rehabilitation program following initial neurological diagnostics. Sessions, lasting 30–40 min, were tailored to the severity of the patient’s condition and conducted by experienced neurologopedic specialists. The therapy addressed aphasia; dysarthria; and partial impairments in swallowing reflexes, phonation, and breathing. Interventions included the use of headphones and multimedia materials for articulation, naming, description, and reading exercises. For motor speech impairments, auxiliary tools such as headphones, microphones, tablets, and labiograms were utilized. Visual aids and common household items supported sensory speech rehabilitation. Pre-developed materials from pedagogical and speech therapy publishers were also employed to enhance speech and cognitive functions. Techniques included auditory processing, memory exercises, spatial relations training, and guided writing and reading activities. All sessions adhered to established standards and procedures in speech therapy [26].

### 2.6. Outcome Measures

Assessments using the below health parameters were conducted as primary outcomes at baseline (M0) and post-intervention (M1), providing a comprehensive evaluation of the intervention’s impact on mood regulation, memory performance, and overall emotional well-being.

#### 2.6.1. UWIST Mood Adjective Checklist (UMACL)

The UMACL measures mood as an affective experience of moderate duration (lasting several minutes or more). It evaluates three core dimensions of affect: Tense Arousal (TA, 9 items), Energetic Arousal (EA, 10 items), and Hedonic Tone (HT, 10 items). The tool consists of 29 adjectives rated on a four-point scale (from “definitely yes” to “definitely no”), reflecting the respondent’s current mood. Validity has been confirmed through factor analysis and correlations with cognitive functions and personality traits. UMACL differentiates individuals with depression based on affect dimensions, with sten scores categorized as low (1–4), average (5–6), or high (7–10). Hedonic Tone (HT) negatively correlates with Tense Arousal (TA) and positively with Energetic Arousal (EA). A high HT with low TA and moderate EA indicates positive mood, while the reverse pattern suggests poor well-being, often linked to depressive disorders. Other configurations, especially if repeated, may indicate mood dysregulation, affecting overall functioning [27,28,29].

#### 2.6.2. Depression Measurement Questionnaire (DMQ)

The DMQ (in Polish, KPD, Kwestionariusz Pomiaru Depresji) is a psychological tool consisting of 75 statements that respondents rate on a four-point scale. The questionnaire assesses key depressive symptoms, including loss of energy, anhedonia, suicidal thoughts, pessimism, and guilt. Results of DMQ are categorized into five scales: Cognitive Deficits and Loss of Energy (CDLE, measures cognitive and behavioral difficulties in 19 items); Thoughts of Death, Pessimism, and Alienation (TDPA, reflects withdrawal and resignation in 15 items); Guilt and Anxiety Tension (GAT, captures emotional issues like guilt and anxiety in 16 items); Psychosomatic Symptoms and Decreased Interests (PSDI, focuses on disruptions in psychophysiological functions in 10 items); and Self-Regulation (SR, evaluates psychological reserves related to emotional and cognitive–behavioral self-regulation in 15 items). The overall score (OS), based on the first four scales (60 items), serves as a general indicator of depression. The OS score of 130 is the cutoff for identifying depressive disorders, with 90% sensitivity and 87% specificity. Elevated scores indicate severe depressive symptoms compared to healthy individuals, with distinct gender-related patterns. For example, in women, higher OS correlates with cynicism and social alienation, while in men, it links to aggression, alcohol abuse, and familial conflicts [30,31].

#### 2.6.3. Benton Visual Retention Test (BVRT)

The BRTV is a clinical tool used to assess visual perception, visual memory, and visuoconstructive abilities. In this study, the test utilized Version C and Method A, where participants viewed each design for 10 s and then reproduced it from memory. The BVRT provides two primary measures: Number of Correct Reproductions is evaluated using an all-or-nothing rule—any error results in a score of 0 for the design. Scores range from 0 to 10 for each test version. Number of Errors in incorrect reproductions are categorized into six types: omissions, distortions, perseverations, rotations, displacements, and relative size errors. This facilitates a qualitative analysis of performance. The total error score can theoretically exceed 24 but rarely does in practice. The BVRT’s validity is supported by several factors, including its sensitivity to age, education level, and correlations with other memory, attention, and executive function tests. It has proven effective in differentiating healthy individuals from those with CNS damage, depression, schizophrenia, mild cognitive impairment, or dementia. Norms are available for individuals aged 5–79 years. High performance (more correct reproductions, fewer errors) indicates better visual perception and memory [32,33,34].

#### 2.6.4. Brain Damage Diagnostic Test (BDDT)

The BDDT (in Polish, DUM, Diagnostyka Uszkodzeń Mózgu) is a nonverbal learning and memory assessment tool designed to detect brain damage-related impairments. It evaluates memory encoding, storage, recognition, retrieval of figural material, and the transfer of recall abilities to motor execution. The test is sensitive to learning changes over a short time, supported by the inclusion of a parallel version. The test set consists of nine 9 × 9 cm cards and five wooden sticks, each 4 cm long. Tasks involve memorizing figures presented on cards and reproducing them using wooden sticks. There are no time restrictions, and the procedure follows standardized protocols. The BDDT is suitable for individuals of all ages, healthy or with neurological conditions, and provides tailored interpretations for children, adolescents with brain dysfunctions, and elderly individuals. Results are evaluated against percentile ranks, with separate norms for individuals with or without higher education. The test’s reliability, first calculated using the test–retest method, showed a coefficient of 0.68, which subsequent studies confirmed and improved upon [35,36].

### 2.7. Statistical Analysis

The results obtained from the study were compiled in an Excel spreadsheet and then exported to STATISTICA v.13.3 (TIBCO Software Inc., Tulsa, OK, USA) for statistical analysis. Continuous quantitative variables (e.g., age) and discrete variables (e.g., the number of comorbidities) were presented in tables as means and standard deviations (M ± SD). If their distributions significantly deviated from the theoretical normal distribution, they were expressed as medians and interquartile ranges—Me [Q1; Q3]. The Kolmogorov–Smirnov test was used to assess the normality of distributions. Since the empirical distributions of most parameters deviated from normality, nonparametric tests were employed in further analysis. The Mann–Whitney U test was used to verify the statistical significance of differences in the mean values of quantitative variables between two independent groups (AIS vs. TBI). The significance of differences between the average values of quantitative parameters in dependent groups (before and after tDCS) was assessed using the Wilcoxon test. Correlations between two parameters were checked by calculating Spearman’s rho correlation coefficient. Statistically significant correlations were illustrated with correlation diagrams. Scatter plots included the parameters of linear regression models. Nominal (e.g., gender) and ordinal qualitative variables (e.g., education level) were presented in contingency tables as counts (n) and percentages (%). The chi-square test was used to assess the significance and strength of relationships between two qualitative variables.

## 3. Results

### 3.1. Participants’ Characteristics

The cohort had a mean age of 49.9 years (SD 17.4), with 67% male participants. Patients from the AIS group were older (59.5 ± 12.4) than TBI patients (40.3 ± 16.5) (*p* < 0.001). Most participants in the AIS group presented with additional comorbidities, such as hypertension (82%) and type 2 diabetes (46%), compared to lower rates of comorbid conditions in the TBI group (HT: 26%; DM: 12%). A majority of participants (73%) had at least a secondary education, with higher education levels more prevalent in the IS group (38%) than the TBI group (24%). The time since the initial event differed significantly between groups. AIS participants were, on average, assessed 8.4 months post-event, while TBI participants were assessed at a mean of 6.1 months post-injury (*p* = 0.034). This variation reflects the different recovery trajectories and rehabilitation timelines associated with each condition.

Among the 100 enrolled patients, the most frequently reported comorbidity was hypertension (25%), followed by atrial fibrillation (12%), type 2 diabetes (5%), and other cardiovascular pathologies (including atherosclerosis, 4%, and prior myocardial infarction, 2%). Hypertensive patients were commonly managed with angiotensin-converting enzyme (ACE) inhibitors (n = 12), beta-blockers (n = 10), and calcium channel blockers (n = 3), according to standard clinical guidelines. Atrial fibrillation was treated exclusively with oral anticoagulants (n = 12), while the few individuals with atherosclerosis (n = 4) received statins and antiplatelet agents. Overall, 6% of participants presented with a previous or current depressive disorder (four in the acute ischemic stroke group, two in the traumatic brain injury group). Of these, four were on selective serotonin reuptake inhibitors (SSRIs), and the remaining two received psychotherapy alone. Anxiety disorders were reported by 5% of the cohort, typically managed with benzodiazepines (n = 3) and/or psychotherapy (n = 2). Similarly, 3% had physician-diagnosed emotional disorders, treated with psychotherapy sessions. An additional 5% were diagnosed with cognitive disorders, receiving multidisciplinary cognitive rehabilitation. Thirteen percent of patients had clinically significant memory impairments, all of whom underwent specialized cognitive training and occupational therapy sessions. Type 2 diabetes was noted in 5% of the sample (n = 5), predominantly controlled with metformin (n = 4) and insulin in one case (n = 1). Four patients (4%) had a history of epilepsy, managed with anti-epileptic medications (e.g., valproate or levetiracetam). Lastly, 34% of participants reported no notable comorbidities. All pharmacological and therapeutic interventions followed established protocols for each clinical condition and were documented at baseline and throughout the study for potential confounding effects on cognitive or emotional outcomes. Table 2 and Table 3 present detailed sociodemographic and clinical characteristics.

### 3.2. Emotional State

Table 4 presents results that the emotional state of participants improved significantly after tDCS intervention, as indicated by transformations in mood parameters measured using the UMACL tool.

Hedonic Tone (HT): defined as the subjective experience of pleasure or unpleasantness; individuals scoring higher on this scale report stronger positive emotions. A statistically significant increase in HT was observed in both groups post-tDCS. In the AIS group, HT increased from 2.5 to 5 stens (*p* < 0.001), while in the TBI group, TH increased from 1.5 to 4 stens (*p* < 0.001). Notably, the improvement in HT was significantly greater in the AIS group compared to the TBI group (Δ sten: 2 vs. 1; *p* = 0.048).

Tension Arousal (TA): associated with stress and anxiety; high scores on this scale indicate increased tension. Both groups demonstrated a significant reduction in TA post-tDCS. Tension levels decreased from 8 to 6 stens in both the AIS and TBI groups (*p* < 0.001 for both). There was no difference between groups noted (*p* = 0.550).

Energetic Arousal (EA): often described as the energy to act; higher scores reflect increased vigor and readiness for action. Post-tDCS, significant increases in EA were observed in both groups. In the AIS group, EA increased from 2 to 3 stens (*p* < 0.001), and in the TBI group, PE increased from 2 to 3.5 stens (*p* = 0.001). Notably, the improvement in EA was significantly greater in the AIS group compared to the TBI group (Δ sten: 2 vs. 1; *p* = 0.048).

### 3.3. Depression Levels

Table 5 presents the medians, quartiles of raw scores, and transformed sten and ten scores for depression levels assessed pre- and post-tDCS. A significant reduction in overall depression levels was observed in both groups, as measured by point scores (*p* < 0.001). Similarly, sten scores reflecting depression levels decreased significantly in both groups post-tDCS (*p* < 0.001). The ten scale, standardized for psychological tests, has a population mean of 50 and a standard deviation of 10. Values between 40 and 60 are considered within the normative range, while deviations outside this range may indicate increased symptom severity. Post-tDCS, depression levels for 50% of patients in both groups fell within the normative range of 40–60, indicating that half of the patients achieved normal levels. The reduction in depression levels was comparable between the AIS and TBI groups (*p* > 0.05).

### 3.4. Memory Performance

Table 6 presents the results of the BVRT, assessing visual memory pre- and post-tDCS in patients from both the AIS and TBI groups. The results showed significant improvements in visual memory performance in both the AIS and TBI groups following tDCS intervention. Correct reproductions increased substantially in the AIS group from a median of 5 to 9 and in the TBI group from 4 to 8 (both *p* < 0.001). Standardized scores improved significantly in both groups, with AIS scores rising from 2 to 3 and TBI scores from 1 to 3 (both *p* < 0.001). Error patterns also improved across groups: omissions, distortions, perseverations, and relative size errors decreased significantly, with the AIS group showing particular reductions in omissions (*p* < 0.001) and misplacements (*p* = 0.012). The overall error index dropped markedly in the AIS group from 7 to 2 and in the TBI group from 9 to 2 (both *p* < 0.001). Additionally, sten scores significantly increased in both groups, from 5.5 to 9 in the AIS group and from 3 to 7 in the TBI group (both *p* < 0.001). These findings underscore the positive impact of tDCS on visual memory and cognitive rehabilitation in patients with brain injuries, demonstrating significant improvements in accuracy and reductions in errors across both groups.

### 3.5. Brain Impairments

The results of the BDDT shown in Table 7 indicated significant improvements in memory and learning in both the AIS and TBI groups following tDCS intervention. The total number of correct responses increased significantly in both groups: AIS from 31 to 39 (*p* < 0.001) and TBI from 28 to 37 (*p* < 0.001). The median scores for correct responses across all six sections of the BDDT improved markedly in both groups, with highly significant *p*-values (all *p* < 0.001). Additionally, the ranked scores showed significant increases, from 8 to 22 in the AIS group and from 8 to 11 in the TBI group (both *p* < 0.001). The percentage of incorrect responses decreased significantly in both groups, indicating fewer errors post-intervention (AIS: *p* = 0.001; TBI: *p* < 0.001). These findings highlight the beneficial effects of tDCS on cognitive recovery, with both groups showing marked improvements in memory performance and error reduction.

## 4. Discussion

The findings of this study confirm the therapeutic potential of tDCS in neurorehabilitation for AIS and TBI patients, with significant improvements observed in emotional states, depression levels, and cognitive performance. These results contribute to a growing body of evidence supporting the role of tDCS in enhancing neuroplasticity and recovery after neurological injury.

Our study’s results align with the work of Bornheim et al. [37], who demonstrated the benefits of tDCS when paired with physical therapy in stroke patients, leading to functional recovery and reduced neurological deficits. The hedonic tone improvements observed in this study, particularly the substantial increases in AIS patients, reflect similar mood enhancements reported in Bornheim’s study, highlighting the role of tDCS in emotional regulation. However, our findings extend beyond motor outcomes by addressing cognitive and emotional recovery, areas that have been underrepresented in prior research.

In contrast to Wang et al. [38], who reported no significant effects of dorsolateral prefrontal cortex (DLPFC) stimulation on short-term memory, our study demonstrated significant gains in both memory accuracy and error reduction using a targeted electrode montage. The discrepancy may arise from differences in electrode placement, stimulation parameters, or the populations studied. While Wang et al. focused on healthy participants, the neurological impairments in AIS and TBI patients may have provided a greater scope for neuroplastic adaptation through tDCS. This contrast underscores the need for condition-specific tDCS protocols, as blanket conclusions regarding its efficacy may not capture its nuanced effects across populations.

The study by Guillouët et al. [39] on aphasia rehabilitation also provides an interesting comparison. They showed significant speech improvements when tDCS was combined with speech–language therapy. While their results focused on linguistic outcomes, our findings of enhanced emotional states and memory functions suggest a broader applicability of tDCS in rehabilitation. This overlap highlights a shared mechanism: the modulation of neuroplasticity and cortical excitability to optimize rehabilitation. Both studies advocate for integrating tDCS into multimodal interventions to maximize its therapeutic impact.

The observed reductions in depression levels resonate with findings by Sampaio-Junior et al. [40], who highlighted the efficacy of tDCS in treating bipolar depression. In our study, depression scores decreased significantly in both AIS and TBI groups, with improvements comparable to pharmacological interventions reported in Sampaio-Junior’s study. These parallel findings suggest that the antidepressant effects of tDCS may be mediated by similar pathways, including enhanced prefrontal cortical activity and modulation of limbic structures.

De Freitas et al. [41] reported similar findings of enhanced episodic memory when tDCS was paired with cognitive training in TBI patients. These results align closely with our observation of significant improvements in BVRT and BDDT scores, further emphasizing the synergistic effects of combining tDCS with rehabilitation exercises. Moreover, Schwertfeger et al. [42] provided a systematic map of evidence for the cognitive benefits of tDCS in TBI patients, reinforcing its value in addressing memory and attention deficits. These findings, when compared to our results, underscore the consistency of tDCS outcomes in cognitive recovery across multiple studies.

However, the outcomes of Miniussi et al. [43] and Xia et al. [44] add complexity to interpreting our results. Miniussi et al. emphasized the variability of tDCS effects based on stimulation parameters and participant characteristics, a point that may explain the differential outcomes between the AIS and TBI groups in our study. For instance, AIS patients exhibited more pronounced improvements in hedonic tone and memory performance, possibly due to differences in baseline neuroplastic potential or the chronicity of their condition compared to TBI patients.

Findings by Xia et al. [44] regarding solute diffusivity modulation offer a novel perspective on tDCS’ underlying mechanisms. While our study did not directly assess such biophysical changes, the observed cognitive and emotional benefits could reflect broader systemic effects of tDCS, including improved synaptic efficiency and neurovascular coupling. Further research should investigate these mechanisms to better contextualize the improvements observed in this study.

The systematic review by Spiroiu et al. [45] explored tDCS’ neurocognitive effects in obsessive–compulsive disorder (OCD), emphasizing its potential in modulating executive function and emotional regulation. While our study focuses on AIS and TBI populations, the reduction in depression levels observed in both groups echoes Spiroiu et al.’s findings, suggesting shared underlying mechanisms across neurological and psychiatric conditions. The observed reductions in tension arousal and increases in hedonic tone in our study may reflect broader effects of tDCS on limbic structures and prefrontal–limbic networks.

Lastly, the work of Lerud et al. [46] and Gibson et al. [47] highlights tDCS’ potential in memory modulation and category learning, respectively. Our findings, particularly the significant gains in BVRT and BDDT scores, align with their conclusions but emphasize the added value of combining tDCS with structured rehabilitation protocols. Furthermore, Quinn et al. [48] demonstrated that tDCS modulates prefrontal–insula connectivity, which may explain the significant improvements in working memory observed in our study. These results support the hypothesis that tDCS induces specific neural network adaptations that facilitate both cognitive and emotional recovery.

In summary, the integration of tDCS with targeted cognitive training may represent a critical step in optimizing rehabilitation outcomes. Pergher et al. [49] highlighted in their systematic review and meta-analysis the enhanced transfer effects observed when tDCS was combined with working memory training, emphasizing the importance of simultaneous interventions in maximizing neuroplasticity and functional improvements. Similarly, Buch et al. [49] proposed consensus-based guidelines for the use of tDCS in motor learning and memory formation, underscoring the need for careful consideration of stimulation parameters, training schedules, and individual patient characteristics to achieve the best outcomes. Based on these insights, the findings of our study suggest that standardized protocols combining tDCS with structured training regimens, tailored to specific cognitive and emotional deficits, could serve as a foundation for developing evidence-based clinical guidelines. Future research should focus on refining these protocols and exploring their applicability across diverse neurological populations to fully harness the synergistic potential of tDCS and rehabilitation training.

### 4.1. Study Limitations

While this study provides compelling evidence for the benefits of tDCS in neurorehabilitation for patients with AIS and TBI, several limitations should be acknowledged. First, the open-label design may introduce bias, as neither participants nor clinicians were blinded to the intervention, potentially affecting the subjective assessments of emotional and cognitive improvements. Second, this study was conceived as an open-label, pilot investigation owing to both the lack of a control group and the modest sample size (n = 100). The lack of a sham control group limits the ability to fully attribute observed effects to tDCS alone, as placebo effects cannot be ruled out. Third, variations in the severity and chronicity of brain injuries among participants may have influenced the outcomes, with heterogeneous baseline characteristics potentially confounding the results. Additionally, the relatively short follow-up period restricted insights into the long-term sustainability of the observed benefits. And last but not least, the higher incidence of certain comorbidities in the AIS group (e.g., hypertension, atrial fibrillation) compared to TBI participants may reflect inherent age or vascular risk factor differences. Future studies should consider a randomized, double-blind design with a sham control, larger sample sizes, and extended follow-up durations to validate these findings and establish more robust evidence for integrating tDCS into standard care.

### 4.2. Practical Implications

The findings of this study suggest that tDCS sessions can be a valuable addition to neurorehabilitation protocols for patients recovering from AIS and TBI. By demonstrating significant improvements in emotional regulation, depression alleviation, and cognitive performance, this study supports the integration of tDCS as an adjunct therapy to enhance traditional rehabilitation methods. The observed benefits in hedonic tone, tension arousal, and memory functions indicate that tDCS can facilitate both psychological and cognitive recovery, potentially leading to improved quality of life and functional independence for patients. Clinicians may consider implementing standardized tDCS protocols tailored to individual patient needs, incorporating targeted electrode placements and optimized stimulation parameters. Additionally, the study highlights the importance of combining tDCS with structured neurologopedic rehabilitation, suggesting that multimodal approaches can maximize therapeutic outcomes. As tDCS is a non-invasive, cost-effective, and well-tolerated intervention, its broader adoption in clinical settings could provide substantial benefits to neurological rehabilitation programs, particularly in resource-limited environments.

## 5. Conclusions

This study highlights the effectiveness of tDCS as a valuable intervention in neurorehabilitation for patients with AIS and TBI. The significant improvements observed in emotional states, depression levels, memory, and learning abilities and brain-related impairments suggest that tDCS has the potential to enhance recovery across cognitive and affective domains. These findings underscore the feasibility of integrating tDCS into standard neurorehabilitation and neurologopedic protocols, offering a non-invasive, safe, and patient-tolerant method for improving quality of life and functional independence.

## Figures and Tables

**Table 1:** Methodology of the electrode placement on the scalp.

Panel [no]	Treatment Time [min]	Current Intensity [mA]	Current Voltage [V]	Number of Sessions [n]	Electrodes’ Location			Electrodes’ Size [cm^2^]	
					Functions	A-tDCS	K-tDCS	A-tDCS	K-tDCS
I	30	1	9	7	Memory	Fp1 F7	O1 F8	5 × 5	7 × 30 7 × 40
II	30–40	1–1.5							
III	30–40	1–1.5			Emotions	Fp2	O2		

Abbreviations: A-tDCS: anodal electrode used during transcranial direct current stimulation; K-tDCS: cathodal electrode used during transcranial direct current stimulation; Fp1 and Fp2: frontal or prefrontal electrodes registering activity mainly from the frontal lobes; F7 and F8: inferior frontal or anterior temporal electrodes registering activity mainly from the orbital, anterior temporal, and lateral frontal areas of the brain; O1 and O2: occipital electrodes registering activity mainly from the occipital lobes; Fp1, F7, O1: left hemisphere electrode locations; Fp2, F8, O2: right hemisphere electrode locations.

**Table 2:** General characteristics of patients in groups differentiated by brain injury type.

Characteristic (Variable)	Total (n = 100)	Type of Brain Injury		*p*-Value
		AIS Group (n = 50)	TBI Group (n = 50)	
**Gender:**				0.523
Women, n (%)	33 (33%)	18 (36%)	15 (30%)	15 (30%)
Men, n (%)	67 (67%)	32 (64%)	35 (70%)	35 (70%)
**Age (years):**				<0.001
M ± SD	49.9 ± 17.4	59.5 ± 12.4	40.3 ± 16,5	40.3 ± 16.5
Me [Q1; Q3]	56 [35; 65]	61 [56; 69]	39 [28; 56]	39 [28; 56]
Min–Max	18–78	18–78	18–72	18–72
**Education:**				0.018
Primary, n (%)	4 (4%)	4 (8%)	0 (0%)	0 (0%)
Vocational, n (%)	6 (6%)	5 (10%)	1 (2%)	1 (2%)
Secondary, n (%)	37 (37%)	19 (38%)	18 (36%)	18 (36%)
Secondary vocational, n (%)	12 (12%)	7 (14%)	5 (10%)	5 (10%)
Incomplete higher, n (%)	10 (10%)	1 (2%)	9 (18%)	9 (18%)
Higher, n (%)	31 (31%)	14 (28%)	17 (34%)	17 (34%)
**Employment status:**				<0.001
Unemployed/student, n (%)	9 (9%)	1 (2%)	8 (16%)	8 (16%)
Retired/disability pension, n (%)	34 (34%)	29 (58%)	5 (10%)	5 (10%)
Employed, n (%)	57 (57%)	20 (40%)	37 (74%)	37 (74%)
**Marital status:**				<0.001
Single, n (%)	32 (32%)	6 (12%)	26 (52%)	26 (52%)
In a relationship, n (%)	68 (68%)	44 (88%)	24 (48%)	24 (48%)
**Type of work:**				0.092
Disability pension, n (%)	19 (19%)	13 (26%)	6 (12%)	6 (12%)
Physical work, n (%)	27 (27%)	15 (30%)	12 (24%)	12 (24%)
Mental work, n (%)	54 (54%)	22 (44%)	32 (64%)	32 (64%)

Abbreviations: n, count; %, proportion; M, arithmetic mean; SD, standard deviation; Me, median (50%); Q1, lower quartile (25%); Q3, upper quartile (75%); Min, minimum value; Max, maximum value; *p*, significance level; AIS, acute ischemic stroke; TBI, traumatic brain injury.

**Table 3:** Clinical characteristics of patients differentiated by type of brain injury.

Characteristic (Variable)	Total (n = 100)	Type of Brain Injury		*p*-Value
		AIS Group (n = 50)	TBI Group (n = 50)	
**Disease duration (months):**				
M ± SD	16.1 ± 6.2	17.3 ± 6.8	15.0 ± 5.3	0.090
Me [Q1; Q3]	12 [12; 24]	12 [12; 24]	12 [12; 18]	
Min–Max	6–36	6–36	12–36	
**Comorbidities:**				
Depressive disorders	6 (6%)	4 (8%)	2 (4%)	0.678
Emotional disorders	3 (3%)	0 (0%)	3 (6%)	0.242
Anxiety disorders	5 (5%)	2 (4%)	3 (6%)	1.000
Cognitive disorders	5 (5%)	2 (4%)	3 (6%)	1.000
Memory impairments	13 (13%)	5 (10%)	8 (16%)	0.552
Cerebrovascular pathologies	5 (5%)	4 (8%)	1 (2%)	0.362
Hypertension	25 (25%)	19 (38%)	6 (12%)	0.006
Atrial fibrillation	12 (12%)	9 (18%)	3 (6%)	0.121
Myocardial infarction	2 (2%)	1 (2%)	1 (2%)	1.000
Atherosclerosis	4 (4%)	4 (8%)	0 (0%)	0.117
Diabetes	5 (5%)	3 (6%)	2 (4%)	1.000
Epilepsy	4 (4%)	2 (4%)	2 (4%)	1.000
No comorbidities	34 (34%)	12 (24%)	22 (44%)	0.035
**Number of comorbidities:**				
M ± SD	0.9 ± 0.8	1.1 ± 0.9	0.7 ± 0.7	0.012
Me [Q1; Q3]	1 [0; 1]	1 [1; 2]	1 [0; 1]	
Min–Max	0–4	0–4	0–2	
**Current rehabilitation:**				
Physiotherapy	68 (68%)	38 (76%)	30 (60%)	0.086
Speech therapy	85 (85%)	39 (78%)	46 (92%)	0.091
Psychological therapy	58 (58%)	16 (32%)	42 (84%)	<0.001
No therapy	2 (2%)	2 (4%)	0 (0%)	0.495

Abbreviations: n, count; %, proportion; M, arithmetic mean; SD, standard deviation; Me, median (50%); Q1, lower quartile (25%); Q3, upper quartile (75%); Min, minimum value; Max, maximum value; *p*, significance level; AIS, acute ischemic stroke; TBI, traumatic brain injury.

**Table 4:** Changes in emotional state pre- and post-tDCS intervention (UMACL).

UMACL	AIS Group			TBI Group		
	Pre-tDCS	Post-tDCS	*p*	Pre-tDCS	Post-tDCS	*p*
**Hedonic Tone (HT) (pts.)**	20 [15; 25]	28 [27; 30]	<0.001	19 [12; 25]	28 [21; 30]	<0.001
**Tension Arousal (TA) (pts.)**	24 [19; 28]	19 [17; 20]	<0.001	25 [22; 29]	19 [17; 21]	<0.001
**Energetic Arousal (EA) (pts.)**	22 [14; 27]	26 [21; 29]	<0.001	23 [19; 29]	28 [22; 30]	<0.001
**Hedonic Tone (HT) (stens)**	2,5 [1; 4]	5 [4; 6]	<0.001	1,5 [1; 4]	4 [2; 5]	<0.001
**Tension Arousal (TA) (stens)**	8 [6; 9]	6 [5; 7]	<0.001	8 [7; 9]	6 [5; 7]	<0.001
**Energetic Arousal (EA) (stens)**	2 [1; 3]	3 [2; 4]	<0.001	2 [1; 4]	3,5 [2; 5]	<0.001

Abbreviations: UMACL, UWIST Mood Adjective Checklist; tDCS, transcranial direct current stimulation; AIS, acute ischemic stroke; TBI, traumatic brain injury.

**Table 5:** Changes in depression level pre- and post-tDCS intervention (DMQ).

DMQ	AIS Group			TBI Group		
	Pre-tDCS	Post-tDCS	*p*	Pre-tDCS	Post-tDCS	*p*
**Overall Score (pts.)**	131 [108; 169]	100 [88; 112]	<0.001	126 [104; 164]	104 [86; 115]	<0.001
**Overall Score (stens)**	8 [6; 10]	6 [4; 7]	<0.001	9 [7; 10]	6 [5; 7]	<0.001
**Overall Score (tens)**	64 [50; 80]	50 [43; 55]	<0.001	64 [54; 81]	52 [46; 57]	<0.001

Abbreviations: DMQ, Depression Measurement Questionnaire; tDCS, transcranial direct current stimulation; AIS, acute ischemic stroke; TBI, traumatic brain injury.

**Table 6:** Changes in memory performance pre- and post-tDCS intervention (BVRT).

BVRT	AIS Group			TBI Group		
	Pre-tDCS	Post-tDCS	*p*	Pre-tDCS	Post-tDCS	*p*
Correct Reproductions	5 [3; 7]	9 [7; 9]	<0.001	4 [2; 6]	8 [5; 9]	<0.001
Standardized Scores	2 [1; 2]	3 [2; 3]	<0.001	1 [1; 2]	3 [2; 3]	<0.001
Omissions	2 [0; 3]	0 [0; 1]	<0.001	3 [1; 5]	0 [0; 1]	<0.001
Distortions	4 [1; 5]	1 [0; 3]	<0.001	4 [2; 6]	1 [0; 4]	<0.001
Perseverations	0 [0; 1]	0 [0; 0]	0.018	0 [0; 1]	0 [0; 0]	0.004
Rotations	0 [0; 1]	0 [0; 0]	0.142	0 [0; 1]	0 [0; 0]	0.002
Misplacements	0 [0; 1]	0 [0; 0]	0.012	0 [0; 0]	0 [0; 0]	0.975
Relative Size Errors	0 [0; 1]	0 [0; 0]	0.006	0 [0; 1]	0 [0; 0]	0.035
Overall Error Index	7 [4;10]	2 [1; 4]	<0.001	9 [5; 14]	2 [1; 6]	<0.001
BVRT (stens)	5.5 [3; 8]	9 [7; 10]	<0.001	3 [1; 5]	7 [5; 9]	<0.001

Abbreviations: BVRT, Benton Visual Retention Test; tDCS, transcranial direct current stimulation; AIS, acute ischemic stroke; TBI, traumatic brain injury.

**Table 7:** Changes in brain damage-related impairments pre- and post-tDCS intervention (BDDT).

BDDT	AIS Group		*p*	TBI Group		*p*
	Pre-tDCS	Post-tDCS		Pre-tDCS	Post-tDCS	
Correct answer I	2 [1; 3]	4 [2; 4]	<0.001	2 [1; 3]	3 [2; 4]	<0.001
Correct answer II	3 [2; 5]	5 [3; 6]	<0.001	3 [2; 5]	5 [3; 5]	<0.001
Correct answer III	5 [2; 6]	6 [5; 8]	<0.001	4 [3; 5]	5 [4; 7]	<0.001
Correct answer IV	6 [4; 8]	8 [6; 9]	<0.001	5 [4; 6]	7 [6; 9]	<0.001
Correct answer V	8 [6; 9]	9 [7; 9]	<0.001	6 [5; 8]	8 [7; 9]	<0.001
Correct answer VI	9 [7; 9]	9 [9; 9]	<0.001	8 [7; 9]	9 [8; 9]	<0.001
BDDT (sum of correct answers)	31 [25; 39]	39 [31; 45]	<0.001	28 [22; 35]	37 [30; 42]	<0.001
BDDT rank	8 [8; 19]	22 [8; 42]	<0.001	8 [8; 10]	11 [8; 25]	<0.001
BDDT (%)	4 [0; 11]	0 [0; 0]	0.001	7 [0; 14]	0 [0; 3]	<0.001

Abbreviations: BDDT, Brain Damage Diagnostic Test; tDCS, transcranial direct current stimulation; AIS, acute ischemic stroke; TBI, traumatic brain injury.

## Data Availability

Data supporting the findings of this study are available from the corresponding author (J.R.) on reasonable request.

## Abbreviations

The following abbreviations are used in this manuscript:

AIS	Acute Ischemic Stroke
BDDT	Brain Damage Diagnostic Test
BVRT	Benton Visual Retention Test
DLPFC	Dorsolateral Prefrontal Cortex
DMQ	Depression Measurement Questionnaire
EA	Energetic Arousal
EEG	Electroencephalography
fMRI	Functional Magnetic Resonance Imaging
HT	Hedonic Tone
OCD	Obsessive–Compulsive Disorder
PET	Positron Emission Tomography
TBI	Traumatic Brain Injury
TA	Tension Arousal
tDCS	Transcranial Direct Current Stimulation
UMACL	UWIST Mood Adjective Checklist
